# Albumin in the Urine

**Published:** 1897-03

**Authors:** Leon L. Solomon

**Affiliations:** Assistant in Chemistry, Kentucky School of Medicine, Louisville


### ALBUMIN IN THE URINE.

By LEON L. SOLOMON, A. B., M. D.

Assistant in Chemistry, Kentucky School of Medicine, Louisville.

Probably no other aid to diagnosis is so regularly and systematically called into use by the careful physician as the examination of the urine.

Indeed, until recently, and before analyses of the blood, sputum, gastric contents, and fæces were shown to throw so much light upon obscure cases, the painstaking doctor felt that he had made use of all means at his disposal to arrive at a sensible conclusion when the urine had been examined. The technique and routine which are most generally pursued by the busy practitioner who undertakes to make this analysis resolve themselves into an examination for the presence of albumin and sugar. This technique, rarely strengthened as it is by a microscopical examination of the specimen, or even by a chemical estimate of the other ingredients of the urine, is open to so many objections and criticisms, and the tests which are ordinarily employed are often so misleading and unsatisfactory, that the wise conclusions which might be drawn from the establishment of the presence or absence of albumin in a given specimen are materially modified. These facts have prompted me to write this paper on Albumin in the Urine. It is my purpose to review briefly the subject of albumin and its origin, and to suggest some changes in the course which is in vogue in urine analysis. Sight must not be lost of the fact that the origin of albumin in the urine may be intrarenal or extrarenal, and if intrarenal, it may be due to any one of three or more probable causes.

First, the structure of the kidney may be so changed by inflammatory processes, or by degeneration, as to permit of the escape of albumin through the thin vascular walls and tubular basement membranes. The presence of albumin in the urine in those diseases which are commonly grouped under the generic name of Bright's disease has its origin in such structural renal change. Albuminuria may, however, be much less serious and of much less import where either the second or third cause is operating.

Second, circulatory disturbances which bring about increased tension in the renal vessels may occasion albumin in the urine. If such circulatory disturbances are kept up long, changes in the renal structure finally follow. Organic heart disease, prolonged external cold and chilling of the surface of the body, physical exercise and fatigue, and disturbances of the vasomotor system, may occasion an increased tension of the renal vessels and thus bring about albuminuria.

Third, hæmatogenic origin. The blood may be so altered in its constitution as to permit the albumin, which ordinarily is not osmotic, to dialyze through the animal membranes of the vessel walls, and pass into the volume of the urine. This is noted at times in cachectic individuals whose kidneys are not the subject of any change. And following the ingestion of chemical agents whose effect is to alter the blood, albumin is almost invariably present. This, then, would suggest how essential it is to go further than a simple albumin estimation, and look for renal derivatives—casts, epithelium, etc —under the microscope, and in fact is a plea for a blood and sputum analysis in conjunction with the urine examination before mistaking a possible anæmia, pure and simple, or an anæmia due to a tuberculosis, for a morbus Brightii. Nor should the albuminuria of extrarenal origin permit us to go astray. It must not be forgotten that urine may leave the kidney free from albumin and be contaminated before it is voided. Such contamination follows when there is present anywhere along the tract of the escaping urine an inflammatory area; whether this is in the pelvis of the kidney, in the ureter, in the bladder, or in the urethra, while urine contaminated with blood, blood coloring matter, fibrin, pus, etc., will surely deceive those whose only task is to find albumin. At the same time, it is necessary to observe still greater care that substances which give reactions similar to those of serum albumin be not mistaken for it—in other words, it is absolutely necessary, when an albumin reaction has been obtained, to determine if such reaction be not due to albumose, to nucleo-albumin, to mucin, to pine acids (oleo-resins), to egg albumin, to hæmoglobin, to peptone, to alkaloids, to urates, phosphates, etc.

After having satisfied ourselves that it is really serum albumin which is present, it is, as a rule, not very difficult to locate the source and cause of the albuminuria, and to this end the microscopical analysis is absolutely necessary. Such an analysis establishes not only the presence or absence of renal derivatives, but of derivatives of the entire urinary tract, from kidney pelvis to meatus urinarius. It shows also any crystals which may be present, and thus offers to us much more satisfactory data from which to derive conclusions and arrive at a sensible diagnosis than is possible even from an exhaustive chemical analysis. No analysis of the urine is, in my opinion, complete unless it is associated with a microscopical examination, and it has long been a question in my mind why life-insurance companies continue to lay so much stress upon the chemical analysis of the urine, and upon albumin, and why they have not long since seen fit to require a careful microscopical report. What right has the examining physician to write a "Yes" after the question "Albumin?" in an insurance application, and thus deny the applicant the privilege of insurance, without modifying it by stating the origin and cause of the albuminuria? This, of course, is impossible unless the examiner looks at the sediment under the microscope. Then, again, I am prone to believe that insurance companies often suffer losses where the examining physician, failing to find albumin, overlooks "a chronic interstitial nephritis." It is a well-known fact that in this form of Bright's disease albumin is often wanting from time to time, and not a few such cases run their entire course, until death ends the chapter, never showing any albuminuria. A microscopical analysis would most likely show such renal derivatives as to make the case suspicious, or at least prompt the doctor to inquire more carefully into the applicant's general condition.

There is no doubt in my mind that in both hospital and private practice, with the present technique and course in urine analysis, errors are constantly being made—that is to say, albumin is being found when it is really not present, and when more reliable tests would prove such albumin to be albumose, mucin, etc. The trouble is, and has been, that with some one of the regular tests—such as the heat test or the nitric-acid test—an albumin reaction is obtained, and the examiner, aside from probably eliminating any possibility of the reaction being due to phosphates or urates, does not take the pains to differentiate the ring or precipitate from an analogous ring or precipitate due to mucin, to alkaloids, or probably to a pine acid, in the specimen, any one of which may be the ingredient of a perfectly normal urine.

This is no argument for a more delicate or more sensitive test. The very delicate tests are apt to be sensitive at the expense of trustworthiness, and it is not sensitiveness which is now needed; albumin, or at least a reaction analogous to that of albumin, has been obtained, and it is only necessary to demonstrate if it is really serum albumin, or if the reaction is due to some other ingredient. Now, for this purpose the time-honored heat test and nitric-acid test are not sufficient. Either of them are most excellent tests, there is no doubt of that, but before pronouncing a reaction to be due to serum albumin other trustworthy corroborative tests must be used and performed in such a manner as to react to albumin and to albumin alone. Such a trustworthy test is the "acetic-acidferrocyanide-of-potassium test," and it should be invariably used in every urine examination. After having found albumin with any one of the ordinary tests, this test should be invariably employed. My plan in urine analysis is as follows: I first employ the heat test and then the nitric-acid test. If albumin is found, I then verify it with the ferrocyanide-of-potassium test. If no albumin reaction is obtained by the heat test and the nitric-acid test, I have recourse to one of the more sensitive tests—as a rule, to the "potassio-mercuric-iodide test," which, in my estimation, is the most sensitive test known. It might be asked, Why is not the heat test, verified by the nitric-acid test, sufficient? I will answer this by saying that the heat test, when employed alone, will not precipitate a possible alkali albumin that may be present, and always precipitates the phosphates (if the urine is neutral or alkaline); and when, as is usually the case, it is employed by first acidulating the urine with a few drops of nitric or acetic acid, albumin, which may be present in a small percentage, is likely to have been rendered acid, and, as we know, acid albumin can not be precipitated by heat. On the other hand, when a considerable percentage of phosphates is present, and not enough acid is added to neutralize all these phosphates, the contained albumin is likely to be alkali albumin, and, as we know, alkali albumin can not be precipitated by heat. Not only is it impossible to estimate, in advance, just how much acid should be added, but when the test is performed in this manner any mucin which may be present is precipitated, and thus renders the diagnosis less certain, while albumose makes us additionally less certain by appearing as soon as the specimen begins to cool. With Heller's nitric-acid test other difficulties beset us. Not only does albumin respond to this test, but the urates, mucin, albumose, the pine acids (oleoresins), and globulin give results quite analogous, and, unless the examiner stops to think that the patient may be taking cubebs or copaiba for an existing bronchitis, he may fail to exclude the oleo-resins (pine acids) which appear in the urine when these remedies are given.

With the "acetic-acid-ferrocyanide-of-potassium test" these difficulties are obviated, and when applied in the proper manner nothing but albumin reacts to the tests, the phosphates, urates, peptones, vegetable alkaloids, and oleo-resins not in the least obscuring the result, because they do not react to the test. It is necessary, though, to thoroughly mix the acetic acid and solution of ferrocyanide of potassium used in the test before adding the urine, as it is the potassium salt which prevents the contained mucin from precipitating. Professor Purdy, on this same principle, has devised an acetic-acid-sodium-chloride test, the latter ingredient preventing the precipitation of the mucin. Finally, it is well to remember that high authorities in urine analysis have seen fit to establish a class of physiological albuminurias, and, while warning against including too many cases in this class, and suggesting that now and then one of such cases may prove later to be a morbus Brightii, maintain that some of them are really physiological, at least functional, albuminurias. With them, it behooves us, nevertheless, to be on our guard, both in diagnosis and prognosis.

In conclusion I might add that the two salient points of my article, and the ones I wish more especially to emphasize, are: First, that more care be exercised in testing for albumin; and, second, that a microscopical analysis accompany every chemical one.—*New York Medical Journal.*

